# Numerical Investigation of CsGeI_3_ Perovskite Solar Cells Employing PANI-Doped Graphene Oxide as a Hole Transport Layer

**DOI:** 10.1021/acsomega.6c10326

**Published:** 2026-10-25

**Authors:** Subaharini Ramalingam, Hariharan Rajasekaran, Thangaraji Vasudevan, Lung-Chien Chen

**Affiliations:** Department of Electro-Optical Engineering, 34877 National Taipei University of Technology, Taipei City 10608, Taiwan (R.O.C.)

## Abstract

Perovskite solar cells (PSCs) have quickly emerged as strong contenders for future photovoltaic technologies, propelled by their outstanding and steadily improving power conversion efficiencies (PCEs). This work utilizes SCAPS-1D to examine and optimize a lead (Pb)-free PSC architecture. An environmentally friendly CsGeI_3_ absorber was incorporated into the device architecture, with polyaniline-doped graphene oxide (PANI:GO) as the HTL and WS_2_ functioning as the ETL. The use of PANI/GO as an HTL represents the primary contribution of this study, providing enhanced charge selectivity and conductivity. Systematic variation of HTL, ETL, and absorber layer thickness was carried out to identify the best device setup. The optimized structure FTO/WS_2_/CsGeI_3_/PANI:GO/Ag achieved a PCE of 30.8%, a *V*_oc_ of 1.14 V, a *J*_sc_ of 31.3 mA/cm^2^, and an FF of 86.4%. These results offer a feasible and eco-friendly pathway toward high-performance Pb-free PSCs, offering important insights that can drive future progress in device engineering and materials development.

## Graphical abstract

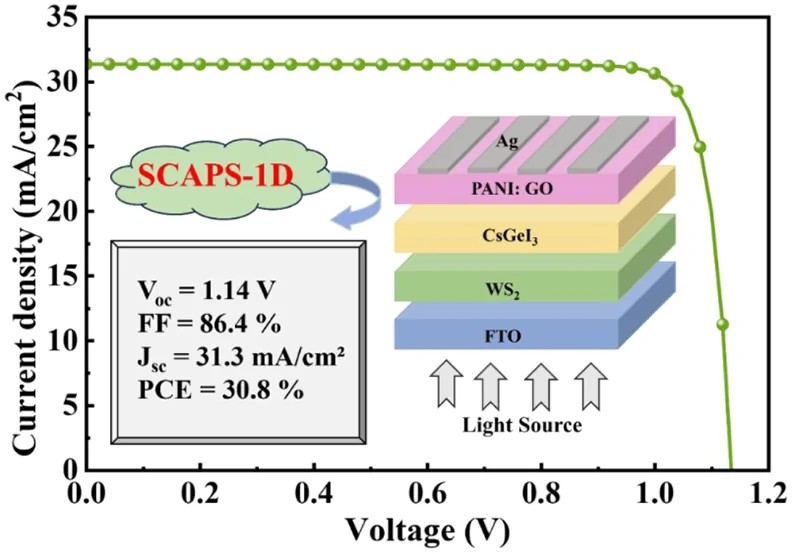

## 1 Introduction

Perovskite solar cells (PSCs) have become one of the most promising technologies in photovoltaic applications of the past decade, with certified PCEs rising from a modest 3.8% in 2009 to over 27% in single-junction Pb-based devices today.^1,2^ This notable advancement is linked to the outstanding optoelectronic properties of halide perovskites (PVSKs), including tunable bandgaps, prolonged charge carrier diffusion lengths, elevated absorption coefficients, low exciton binding energies, and low-cost solution processability.^3^ However, the reliance of high-efficiency absorbers on Pb remains a major obstacle to commercialization, owing to the toxicity of Pb and its associated environmental and health hazards, as well as regulatory restrictions on hazardous materials in consumer electronics.^4,5^ These concerns have driven intensive research toward Pb-free perovskite alternatives, among which tin (Sn)- and germanium (Ge)-based PVSKs have garnered substantial interest because of their favorable bandgaps and similar ns^2^ lone pair electronic configurations to Pb^2+^.^6,7^

Ge-based PVSKs, particularly CsGeI_3_, are of special interest because Ge^2+^ possesses a smaller ionic radius and a stronger stereochemical lone-pair effect than Sn^2+^, which enhances structural distortion, promotes a noncentrosymmetric crystal structure and gives rise to favorable properties such as suitable direct bandgap (∼1.36 eV), high absorption coefficient, and intrinsic p-type conductivity.^8^ These characteristics make CsGeI_3_ a compelling absorber layer for eco-friendlier photovoltaics.^6^ Despite this potential, CsGeI_3_-based devices remain comparatively underexplored relative to their Pb- and Sn-based absorber counterparts and their reported efficiencies still lag behind theoretical expectations, largely due to nonoptimized device architectures, interfacial recombination losses, and selection of charge transport layers.^9,10^

The performance of a PSCe is critically governed by the electron transport layer (ETL) and hole transport layer (HTL) sandwiching the absorber, as these layers dictate charge extraction efficiency, band alignment, and interfacial recombination behavior.^11,12^ An ideal ETL/HTL pairing must offer appropriate conduction and valence band offsets with the absorber to facilitate selective carrier extraction while blocking the opposite carrier, in addition to possessing adequate carrier mobility, chemical stability, and low-cost fabrication compatibility.^13,14^ To identify the optimal configuration for a CsGeI_3_-based device, this work systematically investigates several ETL candidates, such as SnS_2_, ZnSe, C_60_, PCBM, and WS_2_,^12^ and HTL candidates PANI:GO, CFTS, Sb_2_S_3_, and MoO_3_,^15,16–19^ each offering distinct band alignments, carrier mobilities, and defect tolerances that influence overall device behavior.

In this work, a comprehensive numerical analysis is performed on a Pb-free FTO/ETL/CsGeI_3_/HTL device, following the modeling approach and validation strategy adopted in prior numerical studies of Ge-based PSCs.^9,10,20^ Through a screening of ETL and HTL combinations, the optimized FTO/WS_2_/CsGeI_3_/PANI:GO/Ag configuration was identified as the best-performing architecture, achieving 30.8% of PCE, 31.3 mA/cm^2^ of *J*_sc_, 1.14 V of *V*_oc_, and 86.4% of FF, consistent with the high efficiencies reported for WS_2_-based ETL and PANI:GO-based HTL architectures in other Pb-free systems.^12,13^ This shows the strong potential of PANI/GO as a highly conductive polymer-based HTL and WS_2_ as an efficient ETL for CsGeI_3_ PSCs, offering high-performance and environmentally friendly photovoltaic devices.

## 2 Numerical Simulation

The photovoltaic performance of the proposed FTO/WS_2_/CsGeI_3_/PANI:GO/Ag PSCs was numerically investigated using SCAPS-1D (version 3.3.12), a 1D device modeling platform originally developed at Ghent University by Burgelman et al. An ideal approach that has become a standard tool for modeling diverse photovoltaic architectures.^11,15^ The material parameters of the PVSK layer and HTLs are summarized in Table 1, whereas those of the FTO and ETL layers are presented in Table 2. The device architecture enables the extraction of key device metrics, including PCE, *V*_oc_, *J*_sc_, FF, and external quantum efficiency (EQE) spectrum.

**Table 1:** SCAPS-1D Input Material Parameters Used for Simulating the Absorber and Different HTLs

Parameters	CsGeI_3_	PANI:GO	CFTS	Sb_2_S_3_	MoO_3_
Thickness (nm)	600	30	100	30	30
Eg (eV)	1.36	2.76	1.3	1.62	1.2
χ (eV)	3.67	2.02	3.3	3.7	3
ε_r_	18	10	9	7.08	2.5
N_c_ (1/cm^3^)	1.5 × 10^17^	2.2 × 10^18^	2.2 × 10^18^	2 × 10^19^	2.2 × 10^18^
N_v_ (1/cm^3^)	2.8 × 10^18^	1.8 × 10^19^	1.8 × 10^19^	1 × 10^19^	1.8 × 10^18^
μ_n_ (cm^2^/(V s))	20	26	21.98	9.8	25
μ_h_ (cm^2^/(V s))	20	36.2	21.98	10	100
N_A_ (1/cm^3^)	2 × 10^16^	2.0 × 10^17^	1 × 10^18^	1 × 10^15^	1 × 10^–18^
N_D_ (1/cm^3^)	0	0	0	0	0
N_t_ (1/cm^3^)	1 × 10^14^	1 × 10^14^	1 × 10^15^	1 × 10^15^	1 × 10^15^
References	^21^	^22^	^23^	^24^	^25^

**Table 2:** SCAPS-1D Input Material Parameters Used for Simulating the FTO and Different ETLs

Parameters	FTO	WS_2_	PCBM	C_60_	ZnSe	SnS_2_
Thickness (nm)	500	80	50	50	50	50
Eg (eV)	3.5	1.8	2	1.7	2.5	2.24
χ (eV)	4	3.9	3.9	3.9	4.09	4.24
ε_r_	9	13.6	3.9	4.2	9	10
N_c_ (1/cm^3^)	2.2 × 10^18^	1 × 10^18^	2.5 × 10^21^	8 × 10^19^	1.7 × 10^18^	2.2 × 10^18^
N_v_ (1/cm^3^)	1.8 × 10^19^	2.4 × 10^19^	2.5 × 10^21^	8 × 10^19^	8 × 10^16^	1.8 × 10^19^
μ_n_ (cm^2^/(V s))	20	100	0.2	0.08	50	50
μ_h_ (cm^2^/(V s))	10	100	0.2	0.008	20	50
N_A_ (1/cm^3^)	0	0	0	0	0	0
N_D_ (1/cm^3^)	1 × 10^19^	1 × 10^18^	2.9 × 10^17^	1 × 10^17^	1 × 10^15^	1 × 10^17^
N_t_ (1/cm^3^)	1 × 10^15^	1 × 10^15^	1 × 10^15^	1 × 10^14^	1 × 10^14^	1 × 10^12^
References	^26^	^27^	^27^	^27^	^28^	^29^

To realistically capture recombination losses at the layer boundaries, interfacial defect states were introduced at both the ETL/CsGeI_3_ and CsGeI_3_/HTL junctions, as detailed in Table 3. At both interfaces, electrically neutral trap states with a monoenergetic distribution positioned above the valence band maximum were assumed. Electron and hole capture cross sections were assigned at the PANI–GO/CsGeI_3_ interface, while values of 1 × 10^–19^ and 1 × 10^–19^ cm^2^ were applied at the CsGeI_3_/WS_2_ interface, with an integrated interface defect density (IDD) of 1 × 10^10^ cm^–2^ maintained at both junctions. These interfacial parameters were deliberately chosen as interface trap states are well established to degrade both *V*_oc_ and FF in PSCes through enhanced nonradiative recombination.

**Table 3:** Defect Parameters Used for the ETL/CsGeI_3_ and CsGeI_3_/HTL Interfaces in the SCAPS-1D Simulation^a^

Interface	Defect charge state	Electron capture area (cm^2^)	Hole capture area (cm^2^)	Energy level distribution	Band-edge reference energy	Integrated defect density (cm^–2^)
**WS**_**2**_ **/CsGeI**_**3**_	Neutral	1 × 10^–19^	1 × 10^–19^	c	d	1 × 10^10^
**CsGeI**_**3**_ **/ PANI:GO**	Neutral	1 × 10^–19^	1 × 10^–19^	c	d	1 × 10^10^

a Here, “c” is a single parameter, whereas “d” is located beyond the highest eV level.

## 3 Results and Discussion

### 3.1 Device Architecture and Energy Band Alignment

In the simulated FTO/WS_2_/CsGeI_3_/PANI:GO/Ag device (Figure 1a), light is incident through the FTO-coated glass substrate, which acts as the clear front interface with a work function of 4.0 eV and a wide bandgap (E_g_) of 3.5 eV, rendering it optically transparent while providing efficient charge collection. Under equilibrium, dark conditions, the Fermi levels of all layers align, producing band bending at each junction that is governed by the differences in electron affinity and work function between adjacent layers (Figure 1b). This bending is most pronounced across the CsGeI_3_ absorber and its two adjacent interfaces with PANI/GO and WS_2_, which together define the depletion region responsible for charge separation.

**Figure 1:**
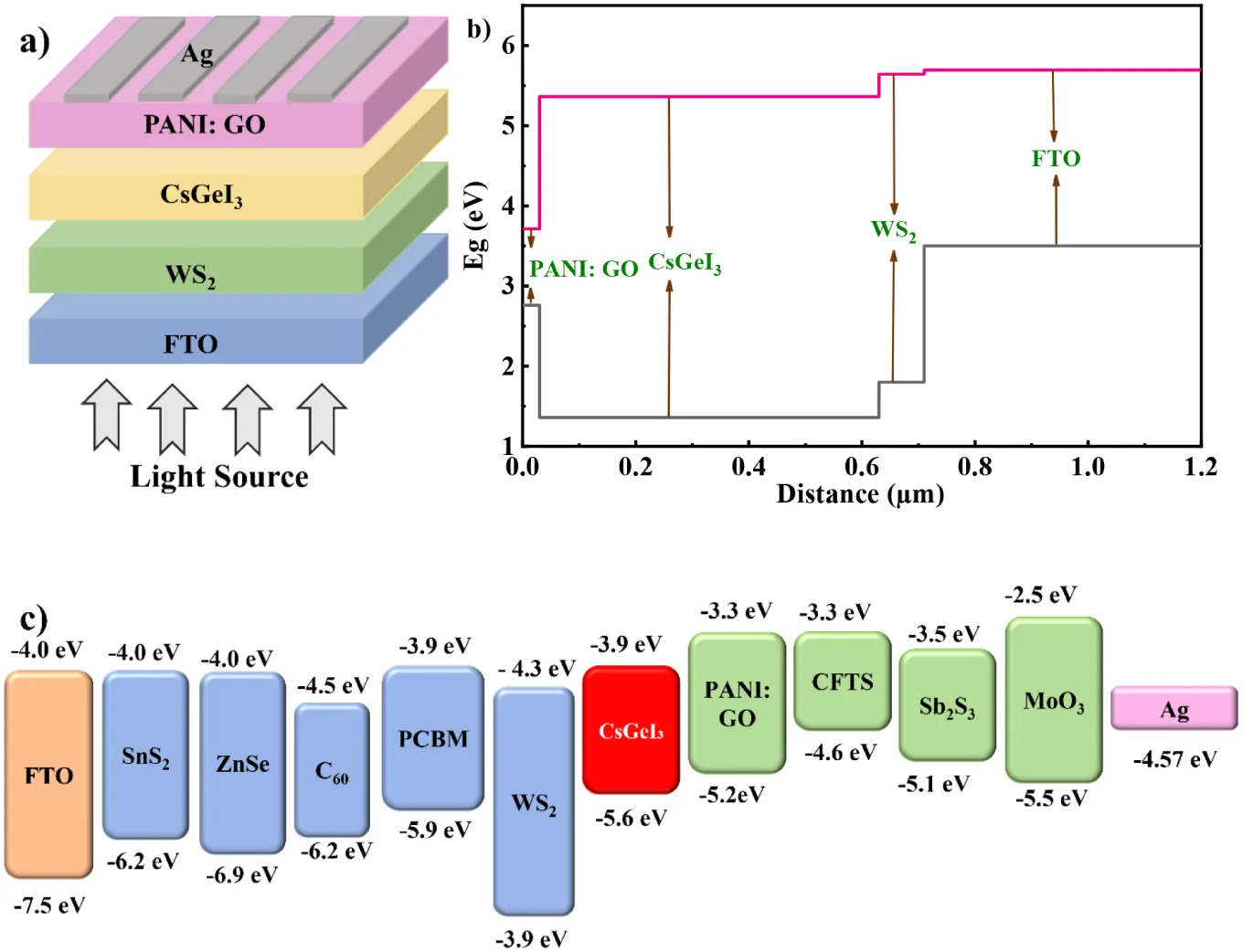
(**a**) Schematic representation of the proposed device structure. (**b**) Spatial distribution of the band edges across the device layers. (**c**) Energy-level alignment of the constituent elements.

The absorber exhibits 3.9 eV of electron affinity and an E_g_ of approximately 1.36 eV, placing its VB edge at 5.6 eV below vacuum, which is near-optimal for efficient solar absorption under the Shockley–Queisser limit (Figure 1c). On the hole transport side, PANI:GO possesses 3.3 eV of electron affinity and an E_g_ of 2.76 eV, positioning its valence band edge at 5.2 eV, and the resulting VB offset of approximately −0.4 eV at the CsGeI_3_/PANI:GO interface (Figure 1b,c) facilitates efficient hole extraction into the HTL while simultaneously serving as a blocking barrier for electrons which suppresses interfacial recombination. On the ETL side, WS_2_ exhibits a relatively high electron affinity of 4.3 eV, resulting in a modest spike-like CB discontinuity of about +0.4 eV at the CsGeI_3_/WS_2_ junction (Figure 1b,c). Since this offset remains within the 0.3–0.4 eV range generally considered not to hinder charge transport, it enables nearly barrier-free electron extraction while still suppressing back-recombination of photogenerated electrons into the absorber.

Upon illumination, electron–hole pairs generated within CsGeI_3_ are separated by the resulting built-in field (Figure 1a), where photogenerated holes migrate through the valence band of PANI:GO toward the Ag back electrode, while light-generated electrons are swept through the conduction band of WS_2_ toward the FTO front contact, whose work function of 4.57 eV completes the device by forming an ohmic contact for electron collection (Figure 1c). This combination of a favorable spike-like CB alignment at the absorber/ETL junction and a blocking VB discontinuity at the PVSK/HTL interface junction is consistent with low interfacial recombination and efficient carrier extraction, both of which are essential to achieving high PCE in this bilayer-free, single-absorber CsGeI_3_ architecture.

### 3.2 Interface Defect Variation across CsGeI_3_/HTL and ETL/CsGeI_3_

The IDD at the CsGeI_3_/HTL junction was modified from 10^10^ to 10^20^ cm^–2^ to evaluate its impact on *V*_oc_, FF, *J*_sc_, and PCE (Figure 2). Three domains emerge as a stable high-performance region at IDD ≤ 10^10^, a sharp transition from 10^11^ to 10^14^ and a saturated low-performance plateau beyond 10^14^. At low IDD, the device delivers optimal performance (1.13 V of *V*_oc_, 86% of FF, 31.3 mA/cm^2^ of *J*_sc_, 30% of PCE) as the interface approaches an ideal, defect-free junction that allows efficient carrier extraction with minimal recombination. Within the transition region, all parameters drop rapidly with the most pronounced decrease occurring between 10^12^ and 10^14^. This behavior is consistent with Shockley–Read–Hall (SRH) recombination, in which defect-induced energy levels create midgap trap levels that facilitate nonradiative recombination. *V*_oc_ drops to 0.99 V due to its logarithmic dependence on interfacial carrier concentration, which makes it highly sensitive to quasi-Fermi level quenching from even moderate increases in trap density. In Figure 2b, FF falls to 75% as heightened interfacial recombination reflects increased series resistance with reduced shunt resistance and distorting the J–V curve at the maximum power point (MPP). *J*_sc_ decreases to 28.7 mA/cm^2^ as trap states increasingly capture carriers before extraction, diverting them from the photocurrent. PCE declines most sharply to 21%, since it simultaneously amplifies the reductions in *V*_oc_, *J*_sc_, and FF.

**Figure 2:**
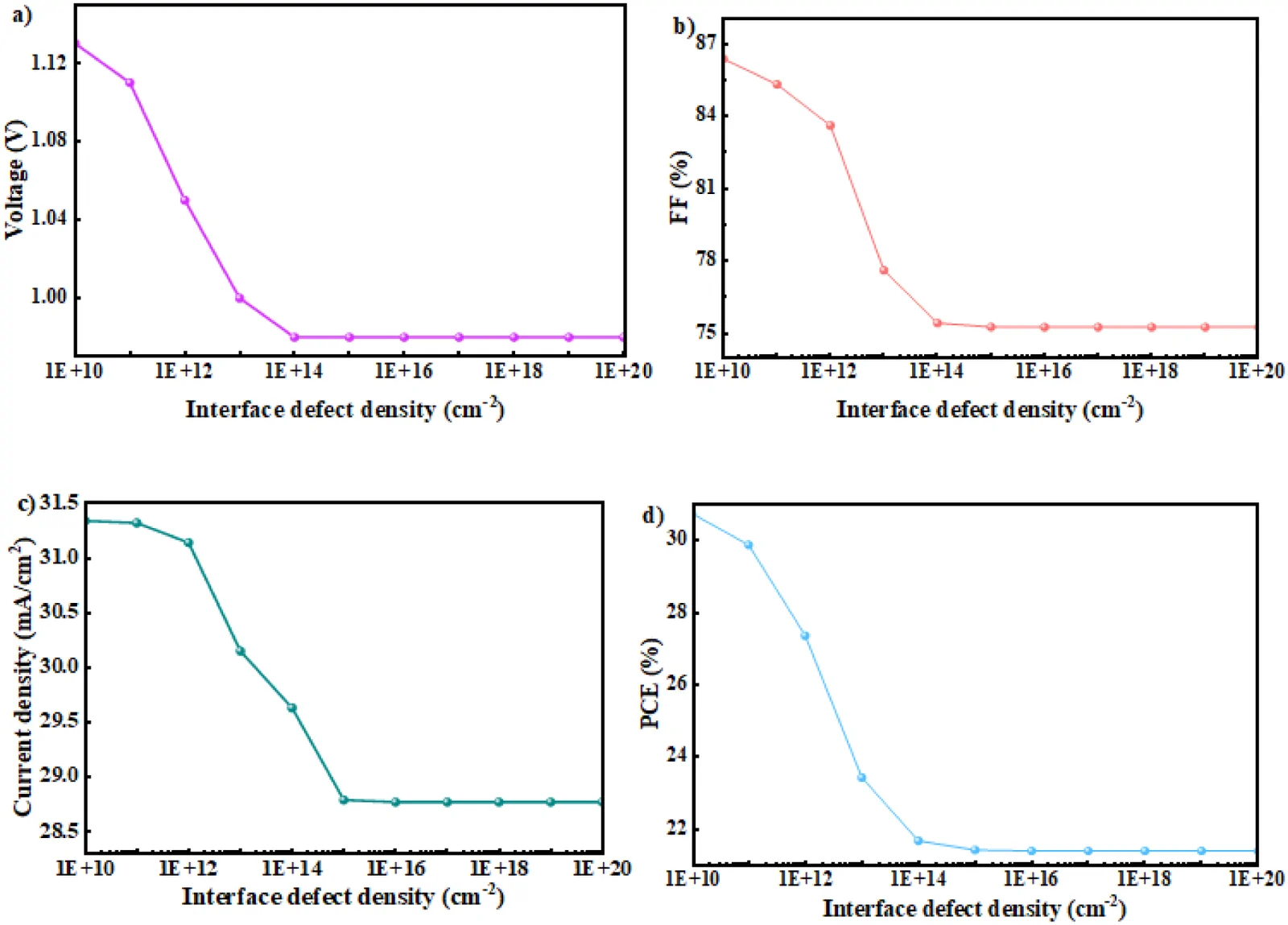
Effect of IDD at the PVSK/HTL interface on photovoltaic performance: (a–d) *V*_oc_, FF, *J*_sc_, and PCE.

Beyond IDD ≈ 10^14^, all parameters from Figure 2a–d saturate and remain essentially constant up to 10^20^, indicating that the interface has reached its maximum recombination capacity, where all carriers reaching the junction recombine and additional defects offer no further loss pathway. At this point, the device transitions from a defect-controlled domain to a saturation plateau. These results establish an interface defect tolerance threshold of approximately 10^10^ to 10^11^, beyond which device efficiency collapses rapidly due to enhanced interfacial recombination. This highlights the critical need for effective interface passivation and high-quality junction formation between the HTL and the PVSK to preserve high photovoltaic performance.

Figure 3 illustrates the effect of changing the IDD at the ETL/CsGeI_3_ interface from 10^10^ to 10^20^ cm^–2^. At the lowest DD of 10^10^ cm^–2^, the device delivers its best performance (*V*_oc_ = 1.13 V, *J*_sc_ = 31.3 mA/cm^2^, FF = 86.4%, PCE = 30.75%), indicating an almost defect-free interface that enables highly efficient charge extraction and transport. As IDD increases, *V*_oc_ steadily declines to ∼1.05 V at 10^12^ cm^–2^ and then more sharply to 0.98 V at 10^14^ cm^–2^, after which it saturates at higher defect concentrations (Figure 3a). This trend is primarily due to increased SRH recombination at the boundary, resulting in a reduction of the quasi-Fermi level gap and thereby lowering the attainable photovoltage. At very low IDD, minor trap-assisted recombination causes a slight FF dip, but the junction quickly restabilizes as trapped charges pin the interface Fermi level, restoring band alignment and giving a small FF recovery (86.3%–85.3%–86%) as shown in Figure 3b. As IDD rises further, the growing trap density increasingly dominates carrier recombination and distorts the local electric field causing FF to steadily decline. Beyond ∼10^17^ cm^–2^, interfacial recombination becomes so severe that it overwhelms any field stabilizing effect, driving the sharp FF drop before it levels off at high IDD once the interface becomes fully saturated.

**Figure 3:**
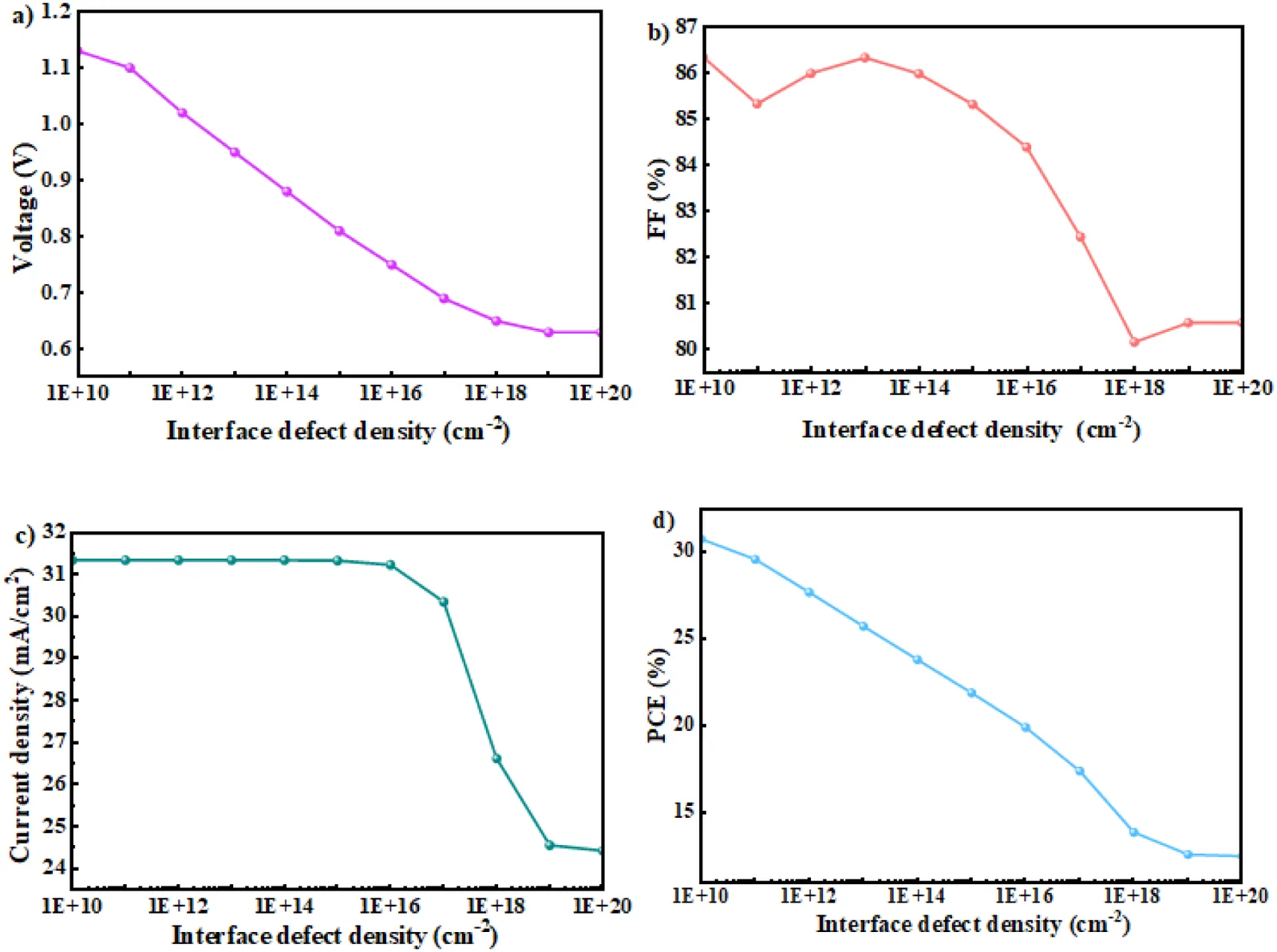
Effect of IDD at the ETL/PVSK interface on photovoltaic performance: (**a–d**) *V*_oc_, FF, *J*_sc_, and PCE.

In comparison, *J*_sc_ stays almost constant up to 10^15^ cm^–2^ (Figure 3c), exhibiting only a slight reduction before decreasing to ∼26.6 mA/cm^2^ at 10^18^ cm^–2^. This suggests that photocurrent collection is relatively less sensitive to low-to-moderate trap densities than voltage-related parameters. For IDD > 10^15^ cm^–2^, *J*_sc_ becomes almost constant indicating that the device enters a region dominated entirely by interface recombination, where additional defects no longer significantly affect carrier collection. As a combined outcome of these parameter changes, the PCE falls from 30.75% at 10^10^ to ∼27.6% at 10^12^ cm^–2^ and then sharply to ∼12.5% at 10^18^ cm^–2^ (Figure 3d). The strong degradation between 10^12^ and 10^14^ cm^–2^ marks this interval as the critical range in which interfacial recombination becomes the dominant loss mechanism, while the subsequent saturation indicates that once this recombination pathway is fully activated, further increases in IDD have minimal additional impact. These results indicate that an IDD below 10^12^ cm^–2^ is essential for realizing improved photovoltaic performance and efficient charge transport of PSCs.

### 3.3 Bulk Defect Variation of CsGeI_3_ Absorber Layer

Figure 4 illustrates the effect of BDD on the photovoltaic response of CsGeI_3_ absorber. Bulk defects act as recombination centers within the absorber layer and significantly influence the charge transport properties. At the lowest DD below 10^13^ cm^–3^, the device approaches the Shockley–Queisser limit (33% for E_g_ = 1.36 eV), while its performance is achieved with a Voc of 1.19 V, an FF of 84.3%, a *J*_sc_ of 31.3 mA cm^–2^, and a PCE of around 31.8%, reflecting efficient charge generation, very low trap-assisted recombination, and effective carrier extraction. As the BDD increases from 10^13^ to 10^20^ cm^–3^, all photovoltaic parameters deteriorate due to the increasing number of defect states that facilitate SRH recombination.

**Figure 4:**
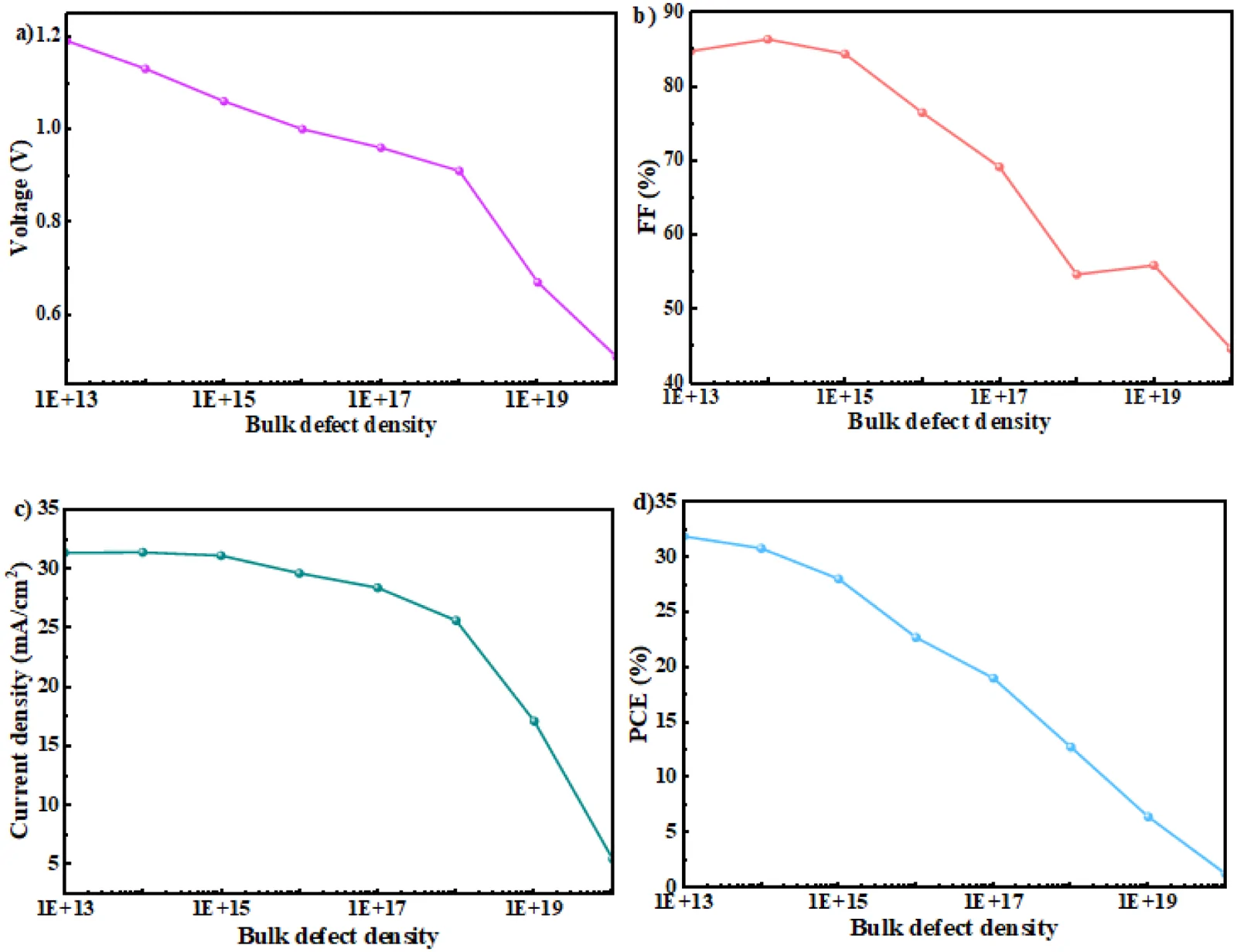
Effect of PVSK bulk defect density (BDD): (**a–d**) *V*_oc_, FF, *J*_sc_, and PCE.

As shown in Figure 4a, *V*_oc_ decreases eventually from 1.18 to 0.91 V as the DD increases as high as 10^18^ cm^–3^, then declines sharply, reaching 0.67 V at 10^19^ cm^–3^ and 0.51 V at 10^20^ cm^–3^. This reduction occurs because the higher concentration of bulk defects enhances nonradiative recombination, thereby reducing the quasi-Fermi level splitting and limiting the maximum attainable photovoltage. The FF presented in Figure 4b exhibits a slight increase from 84.5% to approximately 86% at 10^14^ cm^–3^, suggesting that the influence of isolated defects is negligible at very low concentrations. However, beyond 10^15^ cm^–3^ FF decreases continuously to 45% at 10^20^ cm^–3^ due to enhanced defect-mediated recombination, higher carrier transport resistance and deterioration of the internal electric field, all of which reduce the efficiency of carrier extraction.

The variation of *J*_sc_ in Figure 4c shows that it remains almost constant at around 31 mA cm^–2^ up to a BDD of 10^15^ cm^–3^, indicating that photogenerated carriers are effectively collected when the absorber contains relatively few defects. As the DD increases further, *J*_sc_ gradually decreases to approximately 25.5 mA cm^–2^ at 10^18^ cm^–3^, then undergoes a pronounced drop to 17 mA cm^–2^ at 10^19^ cm^–3^ and 5 mA cm^–2^ at 10^20^ cm^–3^. This behavior reflects the increasing probability of photogenerated electrons and holes recombining through defect states before reaching their respective transport layers. Consequently, the combined reductions in *V*_oc_, FF, and *J*_sc_ lead to a continuous decline in the PCE, as shown in Figure 4d. The efficiency decreases from approximately 31% at 10^13^ cm^–3^ to 28% at 10^15^ cm^–3^, 19% at 10^17^ cm^–3^, 13% at 10^18^ cm^–3^, 6% at 10^19^ cm^–3^, and finally falls to nearly 1% at 10^20^ cm^–3^. The sharp efficiency degradation beyond 10^17^ cm^–3^ indicates that bulk recombination becomes the dominant loss mechanism, severely shortening carrier lifetime and suppressing charge collection. This shows that maintaining a low bulk defect density (≤10^15^ cm^–3^) is essential for minimizing SRH recombination, preserving efficient charge transport and achieving high-performance CsGeI_3_-based absorber.

### 3.4 Analysis of ETL Thickness Variation on Photovoltaic Performance

Figure 5 illustrates the impact of variations in ETL thickness on functional characteristics of the simulated device, comparing five ETL materials such as WS_2_, SnS_2_, C_60_ PCBM and ZnSe across a thickness range of approximately 30 to 150 nm. The operational metrics evaluated include *V*_oc_, FF, *J*_sc_, and PCE, as obtained from numerical investigation. As shown in Figure 5a, *V*_oc_ remains mostly unaffected by ETL thickness for all the materials, indicating that voltage output is primarily governed by band alignment and interface recombination rather than bulk transport-layer thickness. WS_2_ exhibits the highest and most stable *V*_oc_ (∼1.10 V) throughout the thickness range, followed closely by C_60_ (∼1.09 V). PCBM maintains a nearly constant *V*_oc_ (∼1.06 V) but shows a noticeable decline beyond ∼130 nm, likely due to increased resistive losses or altered depletion width at greater thickness. ZnSe remains stable at a slightly lower value (∼1.05 V), while SnS_2_ shows the lowest, though flat *V*_oc_ (∼0.94 V), attributable to a less favorable conduction band offset with the absorber layer.

**Figure 5:**
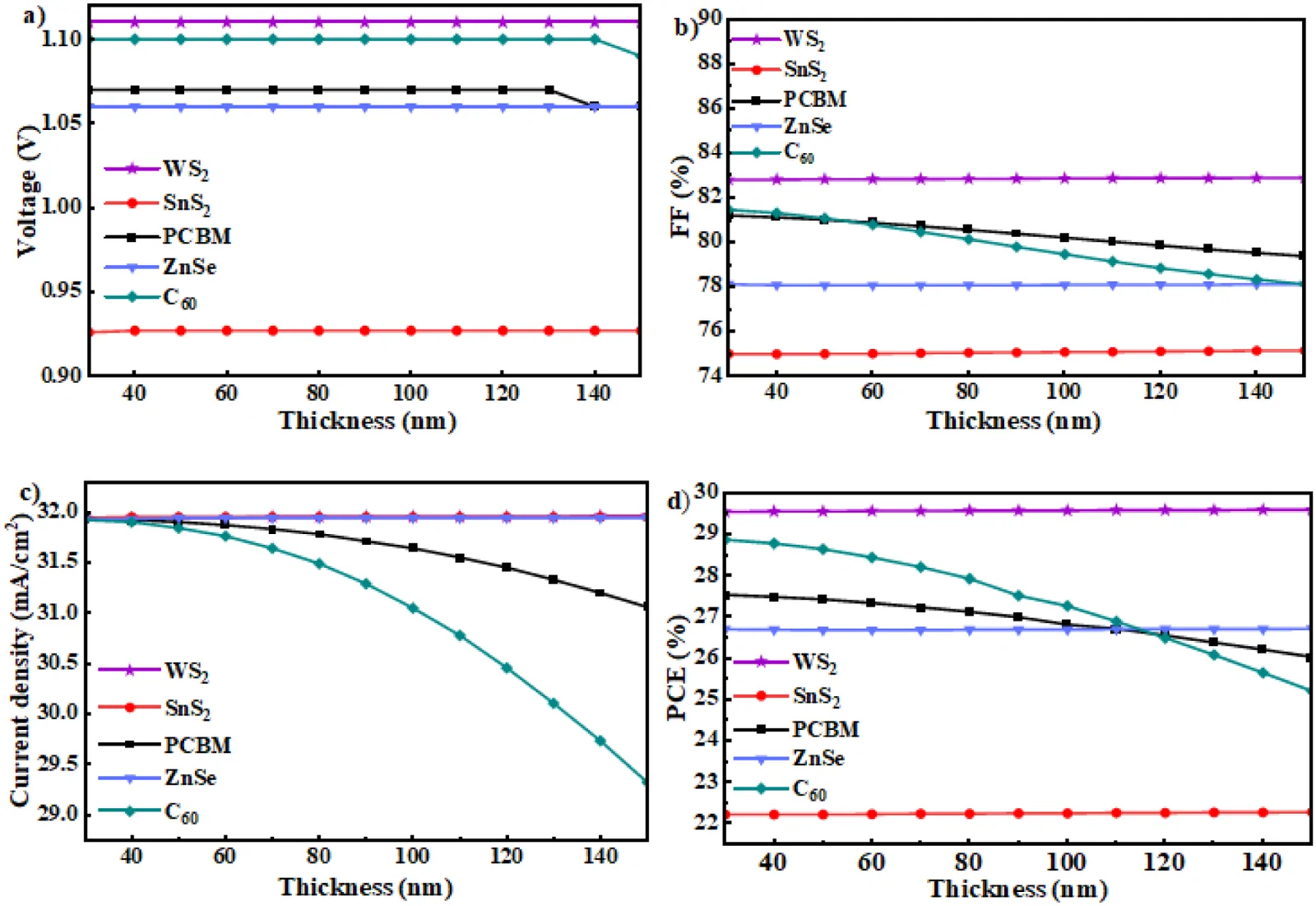
Effect of variations in ETL thickness on functional characteristics: (**a–d**) *V*_oc_, FF, *J*_sc_, and PCE.

The FF presented in Figure 5b, reveals a more pronounced thickness dependence for certain ETLs. WS_2_ and ZnSe display near-constant FF values (∼83% and ∼78%) respectively across the entire thickness range, suggesting minimal additional series resistance is introduced as these layers thicken. In contrast, both C_60_ and PCBM show a steady decline in FF from ∼81% to ∼78% with increasing thickness, consistent with growing transport resistance and carrier recombination losses within the bulk of the ETL. SnS_2_ maintains a flat but comparatively low FF (∼75%) throughout. *J*_sc_, shown in Figure 5c, further highlights the superior stability of WS_2_ and SnS_2_, both of which remain essentially unchanged (∼31.9 mA/cm^2^) regardless of ETL thickness, implying negligible parasitic absorption or transport loss. PCBM exhibits a mild reduction in *J*_sc_ with increasing thickness, while C_60_ shows the most severe decline, dropping from ∼31.8 to ∼29.3 mA/cm^2^ as thickness increases from 30 to 150 nm. This substantial degradation in C_60_ is likely due to increased parasitic optical absorption and reduced effective carrier collection at greater thickness and represents the dominant factor behind its efficiency loss.

Consequently, the PCE in Figure 5d reflects the combined effects of FF, *V*_oc_, and *J*_sc_ variations. WS_2_ delivers the highest and most thickness-independent PCE ∼ 29.3%, underscoring its robustness as an ETL material regardless of layer thickness, a highly desirable characteristic. ZnSe maintains a stable, moderate PCE of ∼26.7%, while PCBM and C_60_ show crossing behavior, where C_60_ initially outperforms PCBM at lower thicknesses, but falls below it beyond ∼110–120 nm due to its steep *J*_sc_ and FF degradation. SnS_2_ consistently yields the lowest PCE (∼22%) across the entire range, reflecting its inherently weaker voltage and FF performance. Among the chosen ETLs, WS_2_ delivers the highest and most thickness-stable *V*_oc_, FF, *J*_sc_, and PCE owing to favorable CB alignment, high electron mobility, and high optical transparency. C_60_ matches WS_2_ only at low thickness and degrades sharply beyond it, making WS_2_ the more fabrication-tolerant ETL for scalable device processing.

### 3.5 Analysis of HTL Thickness Variation on Photovoltaic Performance

Figure 6 illustrates the influence of variations in HTL thickness on the functional characteristics of the device employing four different HTL materials such as PANI:GO, CFTS, Sb_2_S_3_, and MoO_3_. As shown in Figure 6a, *V*_oc_ stays almost unchanged as the HTL layer becomes thicker for all four materials, since *V*_oc_ is mainly regulated by the alignment of energy levels at the HTL/PVSK interface and the associated interface recombination rate, rather than by bulk transport properties, which are largely thickness-independent under open-circuit conditions. PANI:GO and MoO_3_ exhibit the highest and most stable *V*_oc_ values (∼1.11 V), indicating a favorable valence band offset with minimal interfacial recombination, whereas Sb_2_S_3_ shows a slightly lower *V*_oc_ (∼0.99 V) and CFTS shows the lowest (∼0.88–0.90 V), reflecting a less optimal band alignment, whereas the marginal upward trend observed for CFTS with thickness can be attributed to reduced back-contact recombination as the layer becomes less prone to carrier tunneling losses.

**Figure 6:**
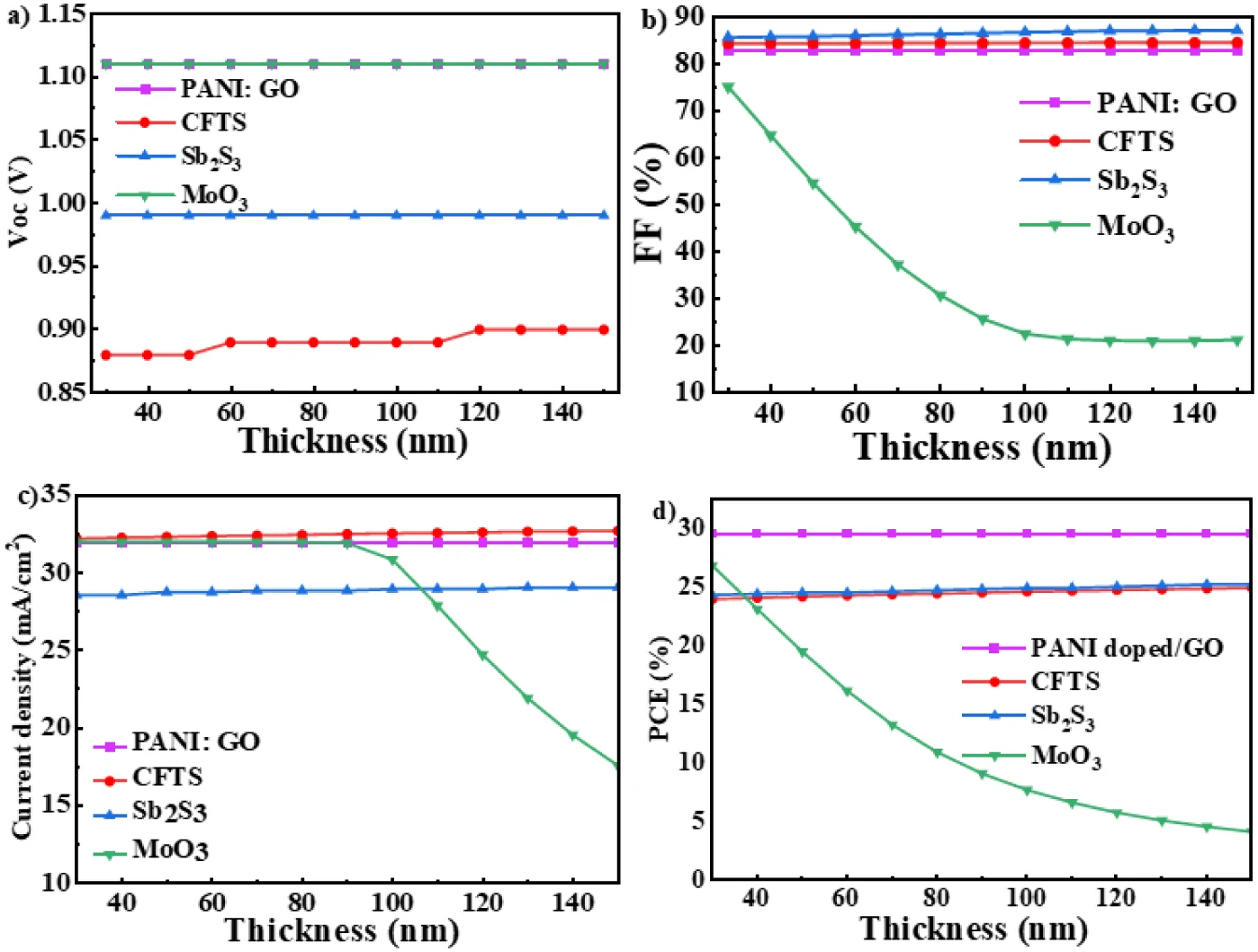
Effect of variations in HTL thickness on functional characteristics: (**a–d**) *V*_oc_, FF, *J*_sc_, and PCE.

In contrast, Figure 6b,c reveals a pronounced degradation in FF and *J*_sc_ for MoO_3_ beyond an HTL thickness of approximately 80–100 nm, while PANI:GO, Sb_2_S_3_, and CFTS maintain nearly constant FF from ∼83–87% and *J*_sc_ across the entire thickness range (∼32 mA/cm^2^ for PANI:GO, CFTS, and ∼32 mA/cm^2^ for Sb_2_S_3_). This behavior arises because FF and *J*_sc_ are strongly responsive to the series resistance and charge transport properties of the HTL. As the layer thickness increases, the resistance of a low-conductivity material rises considerably, since series resistance scales with the ratio of thickness to electrical conductivity; this leads to a substantial voltage drop under operating conditions and a corresponding decline in FF. Simultaneously, photogenerated holes must traverse a longer distance through a resistive medium before reaching the back contact, increasing the likelihood of recombination in transit and thereby reducing *J*_sc_. PANI:GO and CFTS, owing to their comparatively higher hole conductivity and mobility as well as Sb_2_S_3_ are able to sustain efficient carrier extraction irrespective of thickness, whereas MoO_3_, which is conventionally employed only as an ultrathin interfacial buffer layer, suffers from severe transport limitations when scaled to greater thicknesses.

These combined effects are directly mirrored in the efficiency trends shown in Figure 6d. Since PCE is a product of *V*_oc_, *J*_sc_, and FF, the stability of all three parameters for PANI:GO results in an essentially flat and high PCE (∼30%) across the entire thickness range, underscoring its robustness and process tolerance for practical device fabrication. CFTS and Sb_2_S_3_ both show a slight increase in PCE (24–25%), with a stable or marginally rising *V*_oc_ and FF. However, MoO_3_ exhibits a sharp collapse in PCE from over 24% to below 4% as thickness increases, a direct consequence of the resistive losses that dominate their FF and *J*_sc_ behavior. This highlights that PANI:GO offers thickness tolerance due to its favorable band alignment and higher electrical conductivity, which together minimize both interfacial recombination and resistive losses over a wide range of layer thicknesses.

### 3.6 Analysis of Absorber Thickness Variation on Photovoltaic Performance

Figure 7 shows the effect of variation in absorber thickness from 100 to 1500 nm on *V*_oc_, FF, *J*_sc_, and PCE. *V*_oc_ decreases stepwise from ∼1.16 V to ∼1.11 V, while FF, *J*_sc_, and PCE all rise sharply before saturating at larger thicknesses. This apparent contrast is not contradictory but reflects two competing mechanisms, optical absorption and bulk recombination, that respond oppositely to increasing thickness. The rise in *J*_sc_ (Figure 7c) stems from improved light absorption. It fails to fully absorb longer-wavelength photons near the band edge, which penetrate deeper before being absorbed, so increasing thickness captures more of the incident spectrum and generates more electron–hole pairs, until the layer becomes optically thick beyond ∼800 nm and *J*_sc_ saturates. *V*_oc_ (Figure 7a) in contrast, declines because longer carrier transport paths in thicker absorbers increase exposure to defect-assisted SRH recombination across a larger bulk volume, lowering quasi-Fermi level splitting and reflecting the familiar absorption recombination trade-off in thin film photovoltaics.

**Figure 7:**
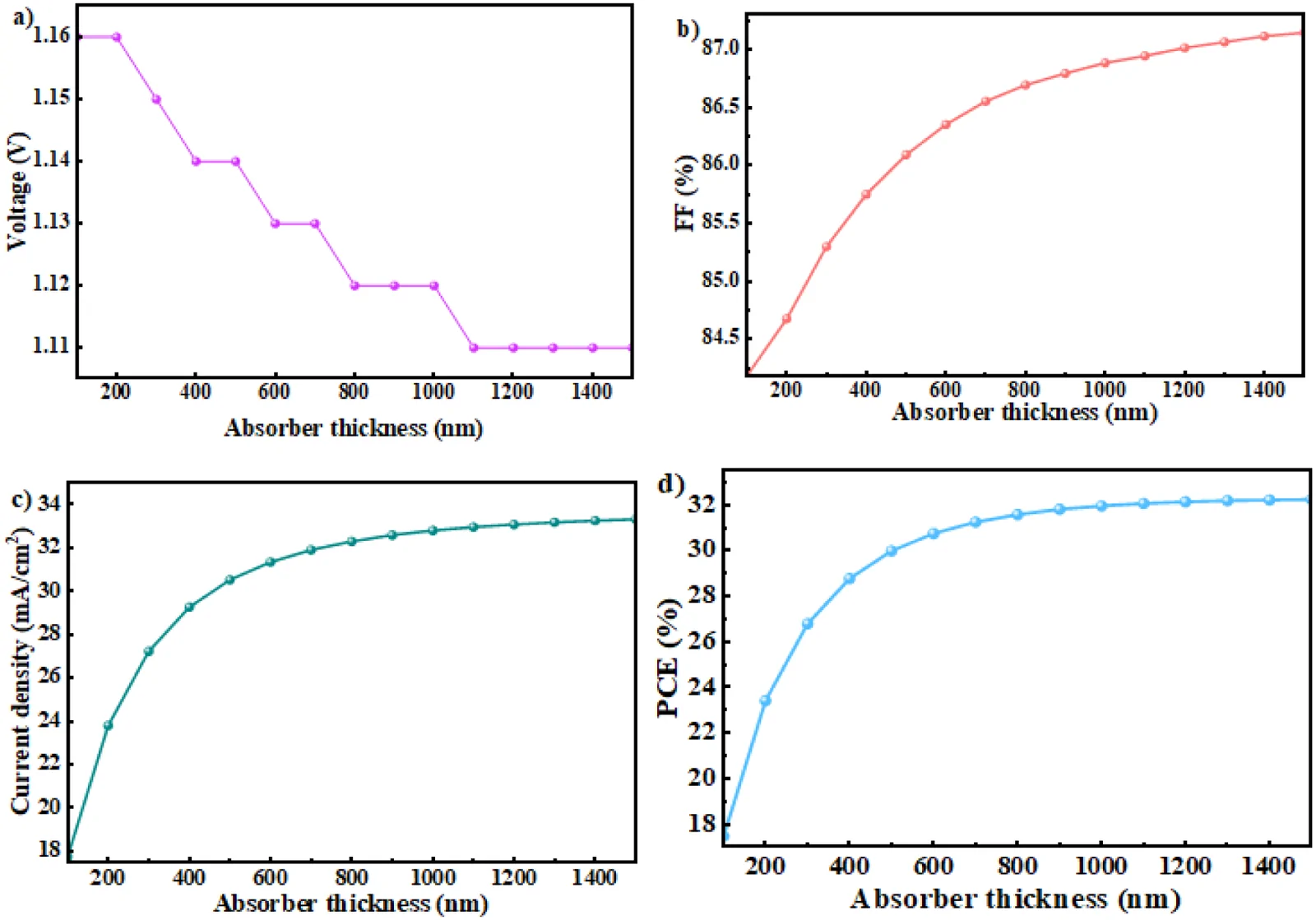
Effect of perovskite (PVSK) thickness variation on device performance: (**a–d**) *V*_oc_, FF, *J*_sc_, and PCE.

FF (Figure 7b) improves with thickness because very thin absorbers suffer from incomplete absorption and a narrow space-charge region, which raise effective series resistance and weaken diode behavior. These effects diminish as thickness increases, until recombination losses at larger thicknesses offset the gain, causing FF to plateau above ∼800–1000 nm. As the fractional increases in *J*_sc_ and FF greatly surpass the minor fractional decrease in *V*_oc_, PCE (Figure 7d) increases significantly, following the trend of *J*_sc_. Above 800 nm, it rises only slightly, peaking at 32.2% around ∼1200–1500 nm, as the minor increases in *J*_sc_ and FF are partially balanced by the ongoing V_oc_ reduction. We chose 600–800 nm as the ideal range, where performance starts to become stable, instead of the peak simulated PCE point. The majority of photogenerated carriers are produced within the initial few hundred nanometers, similar to the diffusion length, thus thicker layers provide minimal advantage while enhancing recombination. This range also maintains performance stability against thickness fluctuations.

### 3.7 Temperature-Dependent Effects on Photovoltaic Characteristics

The influence of working temperature on photovoltaic performance was examined by altering the temperature from 300 to 500 K as shown in Figure 8. As demonstrated in Figure 8a, *V*_oc_ declines from 1.13 to 0.91 V over this range driven by the rapid increase of the current density at reverse saturation (J_0_(T) ∝ T^3^exp­(−E_g_(T)/*kT*)) and the associated rise in intrinsic carrier concentration, both of which accelerate recombination in the absorber and at the junction. Because *V*_oc_ depends on a logarithmic scale concerning the proportion of light-induced to saturation current, even a modest increase in *J*_0_ produces a disproportionately large voltage loss.

**Figure 8:**
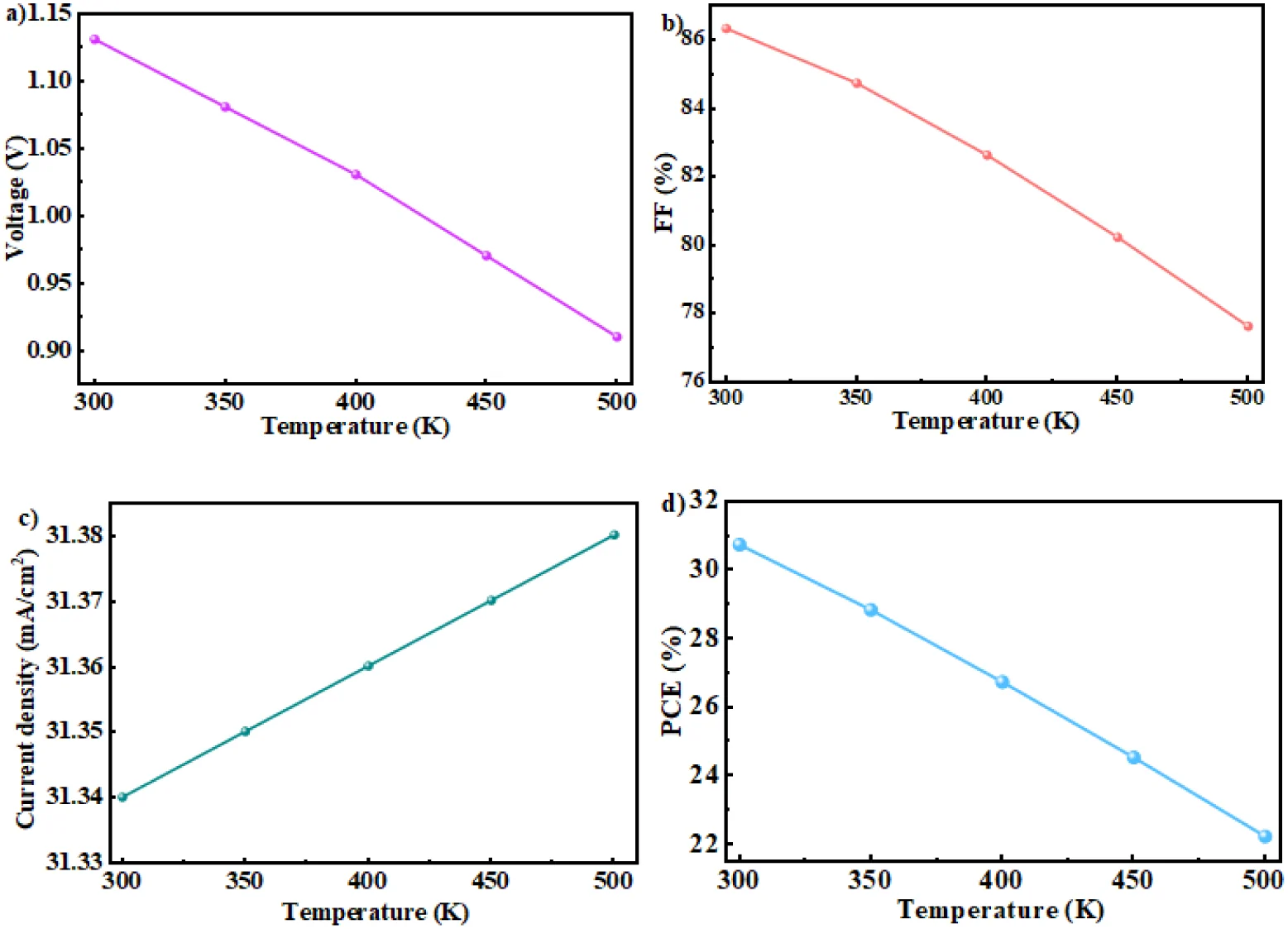
Temperature-dependent photovoltaic performance: (**a–d**) *V*_oc_, FF, *J*_sc_, and PCE.

The FF (Figure 8b) falls from ∼86% to ∼77%, reflecting a different set of mechanisms where intensified phonon scattering decreases carrier mobility and reduces series resistance, while increased recombination at interface defect states lowers shunt resistance and increases leakage current. These opposing resistance shifts flatten the J–V curve and account for the FF decline. *J*_sc_ (Figure 8c), in contrast, rises only marginally from 31.34 to 31.38 mA/cm^2^, as bandgap narrowing extends the absorption edge and enables slightly greater photon collection, a gain that is minor compared to the losses in *V*_oc_ and FF.

Consequently, the PCE (Figure 8d) drops from 30.7% at 300 K to 22.3% at 500 K. Since PCE scales with the product of *V*_oc_, *J*_sc_, and FF, the steep declines in *V*_oc_ and FF dominate the overall trend, despite a small *J*_sc_ gain. This indicates that thermal degradation in this device is mainly controlled by voltage and fill factor losses caused by recombination and resistance rather than by reductions in current, highlighting the need to keep operating temperatures low to ensure stable long-term performance.

### 3.8 Analysis of Photovoltaic and Quantum Efficiency Performance

The J–V characteristics of the optimized device are shown in Figure 9a. The voltage in this case is swept between 0 and 1.2 V. The device sustains an almost uniform photocurrent density of ≈31.3 mA/cm^2^ from 0 V up to almost 1.0 V, indicating excellent charge extraction and minimal recombination losses under reverse-bias. Beyond ≈1.0 V, the photocurrent begins to drop off and the device undergoes a sharp decline between ≈1.05 and 1.15 V before reaching zero current near the *V*_OC_. The extracted photovoltaic parameters are *V*_oc_ of 1.14 V, FF of 86.4%, *J*_sc_ of 31.3 mA/cm^2^, and PCE of 30.8%, reflecting a high-performance solar cell configuration with a steep, well-defined knee in the J–V curve, a signature of low series resistance and high fill factor.

**Figure 9:**
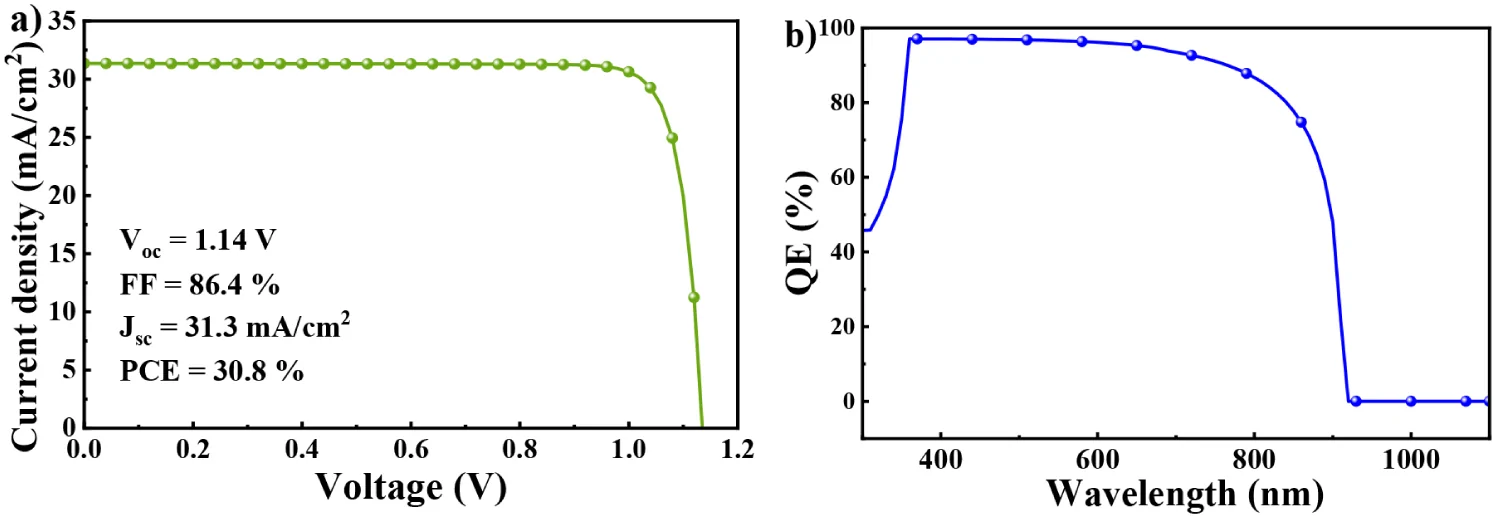
a) J–V curve and b) EQE responses of single-junction PSCs.

The quantum efficiency (QE) in Figure 9b, measured across the wavelength range from 300 to 1100 nm. In the short-wavelength region (≈300–370 nm), the QE rises sharply, reaching a peak of nearly 97%. Across the broad range of ≈370–800 nm, the QE remains essentially flat and high (≈95–97%), indicating that the absorber effectively harvests photogenerated carriers across the majority of the visible spectrum, with a collection efficiency that is largely independent of wavelength. Beyond ≈800 nm, it falls steeply and drops to near zero by ≈900–950 nm, consistent with the absorber bandgap, where photons at the absorption edge with energy beneath the bandgap are no longer absorbed, so QE approaches zero and remains flat out to 1100 nm. The high, flat QE plateau combined with the sharp cutoff correlates well with the high *J*_sc_ obtained from the J–V curve, because an expanded usable spectral window directly results in enhanced photon collection and increased current output.

Table 4 summarizes the photovoltaic performance of previously reported Ge-based PSCs employing different HTLs and ETLs. Various combinations of charge transport materials have been investigated to minimize recombination losses, boost carrier collection, and elevate overall device performance. In comparison, the optimized device proposed in this work demonstrates photovoltaic performance with a PCE of 30.8% together with improved *V*_oc_, *J*_sc_, and FF. These improvements are attributed to the energy-level alignment, efficient charge transport, reduced interfacial recombination, and enhanced carrier collection potential of CsGeI_3_ PSCs.

**Table 4:** Comparison of Numerically Investigated Photovoltaic Performance of the Proposed CsGeI_3_ with Previously Reported Pb-Free PSCes

Device Structure	Voc (V)	Jsc (mA cm^–2^)	FF (%)	PCE (%)	References
FTO/PCBM/MAPbI_3_/PANI:GO/Ag	0.64	21.4	66.26	9.04	^22^
FTO/TiO_2_/CsGeI_3_/Spiro-OMeTAD/Ag	1.18	26.52	88.30	27.63	^30^
FTO/ZnO/CsGeI_3_/Spiro-OMeTAD/Au	0.91	20.84	74.90	14.24	^31^
FTO/C_60_/CsGeI_3_/CuSCN/Au	1.05	22.43	74.80	17.61	^9^
FTO/n-CsGeI_3_/MAGeI_3_/CsGeI_3_/p-CsGeI_3_/Pt	1.30	23.39	88.19	26.85	^32^
FTO/CsGeI_3_/c-Si/Ag Tandem	1.90	17.55	84.90	28.43	^33^
FTO/AZO/Cs_2_AgBiBr_6_/ZnTe/Au	1.50	21.35	83.87	26.89	^34^
FTO/WS_2_/CsGeI_3_/PANI:GO/Ag	1.13	31.30	87.10	30.80	This Work

## 4 Conclusion

This study presents a numerical investigation of a single-junction, Pb-free PSCs with PANI:GO employed as HTL and WS2 as ETL. By systematically optimizing key parameters, including IDD, absorber thickness, and working temperature, the optimized device achieved 30.8% of PCE, 31.3 mA/cm^2^ of *J*_sc_, 1.14 V of *V*_oc_, and 86.4% of FF. These results indicate that the favorable energy band alignment between the CsGeI_3_ absorber and the PANI:GO/WS_2_ charge transport layers effectively suppresses interfacial recombination and promotes efficient charge separation and extraction. Furthermore, the elimination of lead from the absorber composition addresses key environmental and toxicity concerns associated with conventional Ge-based PVSKs, while maintaining excellent optoelectronic performance. All reported values are theoretical predictions obtained from SCAPS-1D simulations under idealized conditions and should be regarded as an upper bound rather than an experimentally demonstrated performance. The proposed architecture therefore represents a promising design candidate for environmentally friendly photovoltaics pending experimental validation. These findings provide valuable theoretical guidance for the design and fabrication of Ge-based PSCs; however, experimental validation and further investigation into long-term stability, scalability, and reproducibility under real-world conditions remain essential steps toward practical deployment.
